# A universal panel of STR loci for the study of polymorphism of the species Canis lupus and forensic identification of dog and wolf

**DOI:** 10.18699/vjgb-24-12

**Published:** 2024-02

**Authors:** A.E. Hrebianchuk, I.S. Tsybovsky

**Affiliations:** Forensic Examination Committee of the Republic of Belarus, Minsk, Belarus; BelJurZabespjachjenne, Minsk, Belarus

**Keywords:** microsatellites, polymorphism, differentiation, identification, Canis lupus familiaris, Canis lupus lupus, wildlife forensic science, микросателлиты, полиморфизм, дифференциация, идентификация, собака домашняя, волк обыкновенный, судебная экспертиза объектов животного происхождения

## Abstract

Commercial panels of microsatellite (STR) loci are intended for DNA analysis of the domestic dog (Canis lupus familiaris) and, therefore, when genotyping the Grey wolf (Canis lupus lupus), most markers reveal significant deviations from the Hardy–Weinberg equilibrium and have a low informative value, which complicates their use in a forensic examination. The aim of this study was to select STR markers that equally effectively reflect population polymorphism in the wolf and the dog, and to create a universal panel for the identification of individuals in forensic science. Based on the study of polymorphisms of 34 STR loci, a CPlex panel of 15 autosomal loci and two sex loci was developed, which is equally suitable for identifying wolfs and dogs. Analysis of molecular variance (AMOVA) between samples revealed significant differentiation values (FST = 0.0828, p < 0.05), which allows the panel to be used for differentiating between wolf and dog samples. For the first time in the forensic examination of objects of animal origin in the Republic of Belarus, population subdivision coefficients (θ-values) were calculated for each of the 15 STR loci of the test system being reported. It was shown that the values of the genotype frequency, when averaged over all studied animals without and with considering the θ-value, differ by three orders of magnitude (3.39 · 10–17 and 4.71 · 10–14, respectively). The use of population subdivision coefficients will provide the researcher with the most relevant results of an expert identification study. The test system was validated in accordance with the protocol of the Scientific Working Group on DNA Analysis Methods. A computational tool was developed to automate the analysis of genetic data on the wolf and dog in the forensic examination; two guides were approved for practicing forensic experts. This methodology is being successfully used in expert practice in investigating cases of illegal hunting, animal abuse and other offenses in the Republic of Belarus.

## Abstract (ru)

Коммерческие панели микросателлитных (STR) локусов предназначены для работы с ДНК собаки домашней (Canis lupus familiaris), в связи с чем при генотипировании волка обыкновенного (Canis lupus lupus) большинство маркеров показывают существенные отклонения от равновесия Харди–Вайнберга и имеют низкий показатель информативной ценности, что осложняет их использование в судебной экспертизе. Целью настоящего исследования стал подбор STR-маркеров, которые одинаково эффективно отображают популяционный полиморфизм волка и собаки, с последующим созданием универсальной панели для дифференциации и идентификации особей волка и собаки в криминалистике. На основе исследования полиморфизма 34 STR-локусов сконструирована панель CPlex из 15 аутосомных локусов и двух локусов половой принадлежности, которая одинаково применима для идентификации волка и собаки. Анализ молекулярной дисперсии (AMOVA) между выборками выявил достоверные значения дифференциации (FST = 0.0828, p <0.05), что позволяет использовать панель для дифференциации образцов волка обыкновенного и собаки домашней. Впервые в судебной экспертизе объектов животного происхождения в Республике Беларусь рассчитаны коэффициенты подразделенности популяции (θ-value) для каждого из 15 STR-локусов разработанной тест-системы. Показано, что значения частот генотипов, усредненные по всем исследованным животным без учета и с учетом θ-value, различаются на три порядка (3.39 · 10–17 и 4.71 · 10–14 соответственно). Применение коэффициентов подразделенности популяции позволит оперировать наиболее достоверными результатами экспертного идентификационного исследования. Предложенная тестсистема валидирована в соответствии с протоколом Scientific Working Group on DNA Analysis Methods. Создан информационно-статистический комплекс для автоматизации обсчета генетических данных волка обыкновенного и собаки домашней в судебной экспертизе, утверждены две методики для практикующих судебных экспертов. Методические разработки успешно применяются в экспертной практике при расследовании фактов незаконной охоты, жестокого обращения с животными и других правонарушений в Республике Беларусь.

## Introduction

According to the statistics of the Ministry of Forestry, in the Republic of Belarus, the Grey wolf (Canis lupus lupus) population has stabilized over the past five years in the range of 1,530–1,630 individuals, which is one of the leading indicators among European countries (Ministry of Forestry of the Republic of Belarus, 2021). Notably, wolf hunting in Belarus is allowed all year long with no sex or age restrictions. At the same time, hunting in protected natural areas and hunting without a permit results in criminal cases and, consequently, the need for forensic examination

According to the Ministry of Housing and Communal Services, about 80,000 stray cats and dogs are exterminated in Belarus every year, and this number is growing, while the exact number of dogs is unknown. Being one of the most common companion animals, the domestic dog (Canis lupus familiaris) has a special status among farm and domestic animals. The active use of dogs by humans in various roles is also reflected in the criminal aspects that accompany the development of society.

The natural history of European wolf (C. lupus lupus) populations has been characterized by a strong reduction in the number of individuals over the past few hundred years (Boitani, 2003). The decline of the population, its fragmentation, and disruption of the gene flow are well-known triggers of genetic impoverishment and increased inbreeding in natural populations, which increase the risk of extinction for wolves as well as for many other species. An example of such a situation was documented for wolves in Italy, where the values of genetic diversity determined by the level of heterozygosity were clearly lower than those in populations from Russia, Alaska, and Canada (Godinho et al., 2011).

Intensive extermination of the wolf can lead to the replacement of the wolf with wolf-dog hybrids. Recently, the problem of hybridization between wolves and free-living dogs in Europe has become a major topic in many research programs (Stronen et al., 2022).

The main difficulty in the genetic differentiation between the wolf and the dog is that DNA markers unique to both the wolf and the dog have not been found. A comparison of dog and wolf genomes showed a similarity of 99 %, which once again confirms their common origin (Freedman, Wayne, 2017).

The study of wolf populations is usually designed according to a typical approach that includes the use of loci recommended by the International Society of Animal Genetics (ISAG) with calculation of statistical indexes of distribution of alleles of the studied loci and assessment of the representation of alleles in the population. Due to the high level of identity of wolf and dog genomes, the ranges of alleles of the loci are very similar Therefore, differentiation of individuals using selected loci becomes possible if genetic differentiation of the studied samples is revealed by statistical processing of genotyping results (Halverson, Basten, 2005; Fan et al., 2016).

Most panels for canine DNA analysis are unsuitable for the study of wolf DNA due to deviation from the Hardy–Weinberg equilibrium and the presence of null alleles in DNA markers. On the other hand, when selecting markers for DNA analysis of the wolf, researchers generally do not take into account the possibility of using the selected markers on an inbred dog population, which precludes the use of these markers to identify a wolf and a dog simultaneously in forensic studies

The aim of this study was to select STR markers that equally effectively reflect the population structure and the polymorphism of the Grey wolf and the domestic dog, followed by the creation of a universal panel for the identification and differentiation of individuals in forensic science

## Materials and methods

Biological objects. Biological samples of the Grey wolf and the domestic dog were obtained legally and are represented by fragments of muscle and cartilage tissues of wolves (n = 103) and samples of blood, hair and saliva of purebred dogs, mestizo dogs and outbred dogs (n = 198). The list of the most represented breeds is reflected in Supplementary Material 11.


Supplementary Materials are available in the online version of the paper: https://vavilov.elpub.ru/jour/manager/files/Suppl_Hrebian_Engl_28_1.pdf


DNA extraction, amplification and genotyping of microsatellite loci. DNA from muscle and cartilage tissues, blood, saliva and hair of wolves and dogs was extracted according to a method based on the release of DNA during the incubation of biological material in a lysis buffer with proteinase K and 0.01 mM dithiothreitol at 37–56 °C. The lysate was subjected to the purification procedure on silica (Boom et al., 1990).

All selected loci were grouped into two test systems: a test system that includes mainly dinucleotide loci recommended by ISAG: AHTk211, FH2054, CXX279, Ren169O18, INU055, AHTh260, INU030, FH2079, FH2848, AHT121, AHTh171, Ren247M23, AHTh130, INRA21, AHTk253, AHT137, Ren54P11, INU005, Ren105L03, Ren64E19, Ren162C04 and Amelogenin sex locus (Radco, Podbielska, 2021); and a test system that includes mainly tetranucleotide loci – FH2096, CPH12, CPH4, FH2004 (Caniglia et al., 2010), FH2016 (Fan et al., 2016), FH2361, PEZ17, FH2328 (van Asch et al., 2010), PEZ16, vWF.x (DeNise et al., 2004), FH2010 (Eichmann et at., 2004), FH2001 (Verardi et al., 2006), VGL3438 (Magory Cohen et al., 2013) and sex loci – DBX and DBY (Seddon, 2005).

Accordingly, the 10 μl PCR reaction volume contained 10 mM tris-HCl, pH 8.6; 25 mM KCl; 2.0 mM MgCl2; 0.2 mM of each dNTP; 0.2–1.0 μM of each of the pair of primers; 0.15 u. DNA polymerase; 1.5 ng/μl BSA; 0.1 % Triton X-100 and 1–20 ng DNA to be analyzed. The polymerase chain reaction was conducted in a thermocycler C1000 (BioRad, USA) using the following program: (1) an initial denaturation step at 95 °C for 5 min; (2) 30 cycles of denaturation at 95 °C (for 30 s), annealing at 60 °C (for 40 s) and elongation at 72 °C (for 1 min); (3) a final elongation at 72 °C for 30 min.

The combination of alleles of each of the samples was detected by electrophoretic separation of PCR products on a 3500 Genetic Analyzer (ThermoFisher Scientific, USA). The size of the detected alleles (in bp) in the studied loci was determined using the Orange 500 bp internal size standards (NimaGen®, The Netherlands) and GeneScan-600 LIZ™ SizeStandard v2.0 (ThermoFisher Scientific, USA). Genotyping was evaluated with GeneMapper ID-X v1.6 software package (ThermoFisher Scientific, USA).

Statistical analysis of the data. Since incorrect species identification distorts calculations based on the analysis of genetic diversity indexes (Galinskayaa et al., 2019), a cluster analysis of genotyping data for wolves and dogs was first carried out for the studied loci. The population structure was determined using the Monte Carlo algorithm according to the Markov chain method using STRUCTURE v.2.3.4 software and Admixture model (Pritchard et al., 2000) with further determination of the true number of clusters by the method of Evanno et al. (Evanno et al., 2005). The burn-in period included 500,000 iterations, followed by the construction of the Markov chain for 1,000,000 iterations for the expected number of groups in the sample, K, from 1 to 10, with six repeats for each value of K. The analysis of clustering in the combined pool of wolves and dogs was performed using the unweighted pair group method with arithmetic mean (UPGMA) and the nearest neighbors joining method (NJ), and the construction of the corresponding dendrograms was carried out using MEGA v.11.0.10 software. To visualize the genetic structure, a multidimensional analysis was performed using the genetic distance matrix following the PCoA method in GenAlEx v.6.5 (Peakall, Smouse, 2006, 2012).

The calculation of frequencies of alleles of STR loci and values of the observed (НO) and expected heterozygosity (НЕ), as well as evaluation of deviation from Hardy–Weinberg equilibrium was performed using Cervus v.3.0.7 software package (Kalinowski et al., 2007). To identify possible errors in the interpretation of genetic profiles, null alleles, and PCR artifacts, an analysis was carried out using Micro-Checker v.2.2.1 software (van Oosterhout et al., 2004).

Analysis of molecular dispersion and estimation of inbreeding coefficients were performed using Arlequin v.3.5.1.3 software (Excoffier et al., 2005). Analysis of assignment of the individual to the sample pool (Assignment Test) based on the selected loci was performed using GenAlEx v.6.5. Polymorphism information content (PIC) of the selected STR loci was calculated using Cervus software v.3.0.7.

Alleles sequence determination. To identify possible microvariants of the sequence, as well as to perform tandem determination of alleles, which is a common practice in forensic science, the primary structure of alleles was determined by Sanger dideoxy sequencing (Sanger et al., 1977). Nucleotide sequences of alleles of each STR locus and sex loci were determined in the forward and reverse directions. Sequencing was performed on a 3500 Genetic Analyzer and was conducted with the BigDye® Terminator v.3.1 Cycle Sequencing Kit (ThermoFisher Scientific). Comparative analysis of allele sequences of the studied loci was carried out in BioEdit v.7.0.5.3 (Hall et al., 2011). The sequences of each locus with the minimum and maximum allele molecular sizes were deposited in the GenBank database (Benson et al., 2005) with assignment of corresponding access numbers.

## Results and discussion

**Fig. 1.**
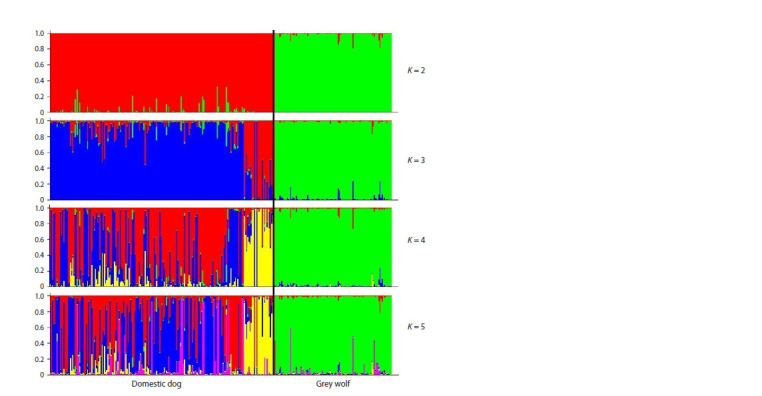
Results of cluster analysis of wolf and dog samples, performed in STRUCTURE software, for the most probable value of the number of genetic clusters K = 2–5, sorted by samples.

A posteriori analysis of the STRUCTURE results for the combined pool of the wolf and dog genotypes revealed the maximum value of the test statistics ΔK at K = 2, which indicates the presence of two genetic clusters in the analyzed sample of animals: the Grey wolf (green cluster) and the domestic dog (red cluster) (Fig. 1, Supplementary Material 2). When determining the population structure of samples of wolves and dogs separately from each other, the cluster formed by the wolf samples remained homogeneous; the absence of clustering within the wolf population using STR loci was previously shown by researchers in Europe (Aspi et al., 2006; Sastre et al., 2011; Ðan et al., 2016).

For a separate analysis of STRUCTURE in the samples of dogs, four groups were formed: purebred dogs (n = 78) and three breed groups: Molossians (n = 32), Samoyeds (n = 66) and Shepherd dogs (n = 22). The maximum value of ΔK at K = 2 indicated the formation of two clusters (Supplementary Materials 3 and 4), one of which corresponds to Shepherd dogs, whereas the breed groups of Samoyeds and Molossians were not separated and, moreover, did not differ from the group of outbred dogs

At the same time, the analysis of clustering in the combined pool of wolves and dogs by the methods of UPGMA and NJ with construction of corresponding dendrograms revealed four clusters of different hierarchical levels in the sample of dogs. The greatest similarity was observed between the breed group of Molossians and outbred dogs. They are adjacent to the breed group of Samoyeds, and this entire group is separated from the group of Shepherd dogs. Since both dendrograms showed a similar structure, Supplementary Material 5 shows only the dendrogram built according to the UPGMA method (with bootstrapping for 10,000 permutations). It should be noted that a similar pattern of clustering of dog breeds in the study of SNP markers was obtained by other authors (Parker et al., 2017).

Analysis of the population structure of wolves and dogs showed a strong genetic differentiation between them with the average values of the cluster membership coefficient Q of 0.984 and 0.981, respectively. The data obtained on differentiation of the wolf and the dog by 34 STRs are in good agreement with the data of Korablev et al. (2021). The level of differentiation between dog breeds was much lower than between the wolf and the dog. Q values varied from 0.457 to 0.495 for Molossians, from 0.451 to 0.476 for Samoyeds, and from 0.740 to 0.757 for Shepherd dogs.

**Fig. 2.**
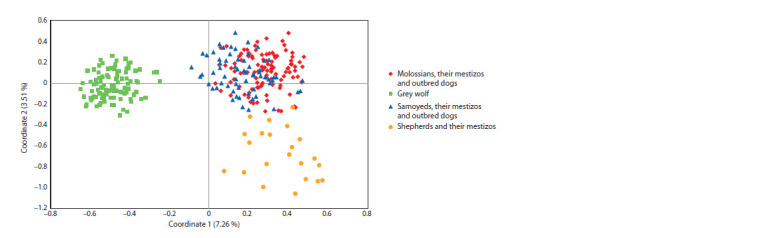
The diagram of the results of PCoA analysis based on the matrix of paired FST values for samples of the Grey wolf and domestic dog

The results of the cluster analysis are consistent with the multivariate genetic distance matrix analysis (PCoA method), which also shows a strong differentiation between the wolf and dog samples (Fig. 2). Similarly, Shepherd dogs form a separate group. The latter outcome may be explained by selection differences or may be a consequence of the high heterogeneity of the samples of Molossians and Samoyeds, which have a common historic origin (over 20 different breeds), while the sample of Shepherd dogs included only purebred German Shepherds.

In total, 405 alleles were identified in the sample of wolves and domestic dogs using the two test systems. All the loci were polymorphic and had from 5 (FH2096) to 26 (FH2361) alleles per locus. The average number of alleles per locus in all samples was similar, amounting to 9.402 ± 0.617 (Table 1). The Shepherd dog was an exception, with the mean value per locus in the range of 5.824 ± 0.328, which could be a consequence of the small sample size (n = 22).

**Table 1.**
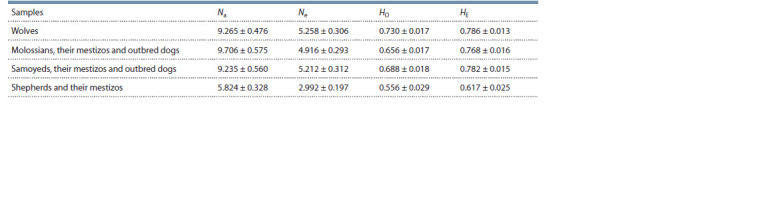
Average values of the level of polymorphism of the studied loci in samples of wolves and three historical breed groups of dogs Na – number of alleles per locus; Ne – effective number of alleles per locus; HO – observed heterozygosity; HE – expected heterozygosity

The highest observed and expected heterozygosity rates were obtained for the wolf sample, at 0.730 and 0.786, respectively (see Table 1). In dog samples, lower values of expected heterozygosity compared to observed heterozygosity are a potent indicator of the presence of inbreeding resulting in synthetic selection and genetic drift, which can irreversibly remove alleles from the population, leading to significantly reduced diversity (Galinskayaa et al., 2019). Although mutations counteract the genetic drift, it is difficult to achieve a balance of genetic processes in dog breeding due to exclusion of individuals with identified mutations in a particular trait. The highest values of heterozygosity and the highest effective number of alleles were found in the wolf sample, which indicates natural development and presence of mutation-drift balance in the natural population.

The analysis of the profiles of dinucleotide loci requires special attention since fragments that do not belong to true alleles and are stutter products can be present on electrophoregrams. The percentage of stutter products usually increases with the length of the allele. Based on the analysis of the genotypes using Micro-Checker software, three of the 20 dinucleotide markers (INU055, Ren169O18, and Ren64E19) showed a high probability of genotyping errors and were excluded from further analysis

The analysis of the distribution of alleles for two loci (AHT121 and AHTk211) showed the presence of a large number of null alleles in three samples, including the sample of the wolf. AHT137 and INRA21 loci showed a high null alleles content in two samples of dogs, 8 loci (AHTh130, AHTh260, CXX279, FH2848, Ren105L03, Ren162C04, Ren247M23, and Ren54P11) showed a rather high null alleles content (from 5.7 % in the sample of Molossians at the FH2848 locus to 12.6 % at the CXX279 locus in the sample of Shepherd dogs). These loci were also excluded from further work on the design of a universal forensic panel.

When analyzing the pattern of frequency distribution of alleles in specific loci, special attention was paid to loci with a significant predominance of major alleles. Differences in the frequencies of major alleles can be very instrumental for differentiating wolves and dogs by microsatellite analysis; however, a significant predominance of one allele can affect the level of identification confidence. Due to the pronounced dominance of major alleles, INU030, INU005, AHTk171 and AHTk253 loci were excluded from further analysis

Most of the studied loci in the samples of wolves and dogs conformed to the Hardy–Weinberg distribution ( p > 0.05), including AHTh171, AHTk253, CPH12, CPH4, FH2001, FH2004, FH2010, FH2016, FH2096, FH2328, FH2361, INU005, INU030, INU055, PEZ16, PEZ17, Ren169O18, Ren64E19, VGL3438 and vWF.x. Two loci, FH2054 and FH2079, deviated from the equilibrium ( p = 0.005) in all studied samples; however, when using the Bonferroni correction, p-values ceased to be statistically significant. In 12 loci, a statistically significant deviation from the Hardy–Weinberg equilibrium was revealed in at least one sample, which may reflect the manifestation of loci in the studied samples and can be explained by the presence of null alleles

The allele fixation index (FST) ranged from 0.025 (FH2001) to 0.158 (CPH4), with an average value of 0.077 for all loci. The highest FIS values were obtained for the FH2079, FH2096, and CPH12 loci (0.221, 0.174, and 0.162, respectively). Overall, for a panel of 15 selected loci, inbreeding indexes (FIS and FIT) were 0.103 and 0.172, respectively (Table 2).

**Table 2.**
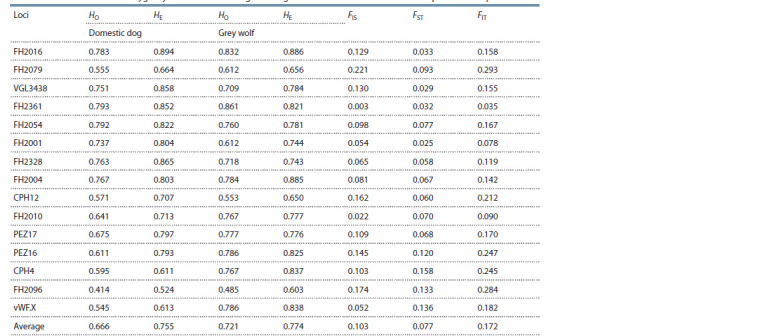
Mean values of heterozygosity for the wolf and dog and Wright F-statistic values for the total sample of Canis lupus HO – observed heterozygosity; HE – expected heterozygosity; FIS − inbreeding index of individuals within the sample; FST – allele fixation index; FIT – inbreeding index of individuals in the total sample.

FIS values in the sample of wolves in most of the studied loci showed values approaching zero, which, together with high heterozygosity values, can indicate the presence of panmixia in the population (Galinskayaa et al., 2019).

A correct assignment of a sample to a Grey wolf or a domestic dog can be of crucial importance in a forensic investigation. Analysis of molecular variance (AMOVA) was performed to assess the possibility of differentiation between a wolf and a dog using selected microsatellite loci. The AMOVA results showed that the percentage of variation between wolf and dog samples was 8.28 %, while within samples it was 91.72 %. Variance components in the population were significant for all studied loci (Supplementary Material 6), which indicates differentiation of the wolf and dog samples. The vWF.x, FH2096, and CPH4 loci accounted for 21.11, 19.34, and 14.40 % of inter-sample genetic variability, respectively, while FH2079 and FH2016 showed the lowest inter-population variability (2.45 and 2.20 %, respectively).

According to Wright’s interpretation (Wright, 1978), the range of FST values from 0.15 to 0.25 indicates moderate differentiation. At the same time, values in the range of 0.00– 0.05 indicate a weak but noteworthy difference between the samples. Since hypervariable markers with a large number of alleles can have significantly lower FST values than markers with a small number of alleles, it is more important to detect significant genetic differentiation between wolves and dogs in the totality of the selected STR loci (Hedrick, 2000). Betweensample AMOVA analysis revealed significant differentiation between the wolf and the dog (FST = 0.0828, p < 0.05).

**Fig. 3.**
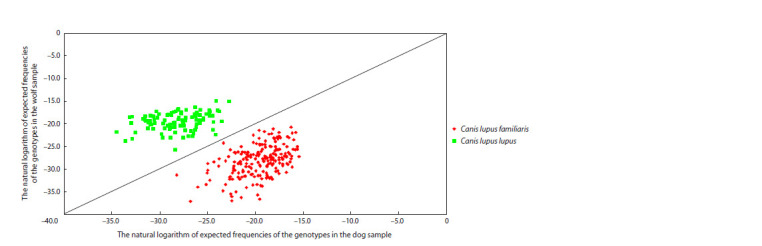
Graph of the genetic assignment of the genotype to the study sample (graphical interpretation of the Assignment test).

The significance of the differentiation of the wolf and the dog using the selected loci can be illustrated by the analysis of assignment of a definite sample (Assignment test). The analysis is based on the calculation of the probability value of the presence of the genotype of a certain individual in the sample from which it was selected, and its comparison with the probability value of the same genotype in another sample. Based on these calculations, an individual belongs to the sample for which it has the highest probability (Fig. 3).

The calculation of true genetic affiliation to the sample showed a high consolidation of wolves and dogs, with 100 % of all studied animals genetically assigned to their own cluster. At the same time, we observed a large difference in the moduli of natural logarithms of expected frequencies of the genotypes when they belonged to their own versus an alternative sample; for wolves, the difference was on average 8.722, and for the domestic dog, it was 8.584. The high values of the difference between the moduli of the logarithms of the expected genotype frequencies confirm successful differentiation of the Grey wolf and the domestic dog using the proposed loci.

An important criterion in forensics is adequate interpretation of the value of the reliability level of an identification study. To calculate the probability of accidental matching of genotypes, an appropriate reference database and correct application of a conservative calculation procedure are required (Buckletone et al., 2016). The study of genotype pools of samples of wolves and dogs made it possible to calculate the frequencies of occurrence of alleles and corrections for the genetic subdivision of the populations of wolves and dogs living in Belarus

A complex social organization of the wolf pack, along with targeted dog breeding, lead to formation of structured populations of these animals. Therefore, in order to obtain the most reliable conclusions, using frequency characteristics of the loci of specific subpopulations would be the preferred approach in an identification study, but this is hardly possible in practice in forensic examination of objects of animal origin. An alternative solution is to introduce a subdivision coefficient (θ-value) into the calculation of the frequency of the genotype, which takes into account the presence of structured populations (The Evaluation…, 1996; Buckleton et al., 2006).

As can be seen from Table 3, the values of genotype frequencies averaged over all studied animals and calculated with the inclusion of subsequent studied loci of the test system without and with taking into account the θ-value differ by three orders of magnitude (3.39 · 10–17 and 4.71 · 10–14, respectively). This suggests that not factoring in the θ-value in the identification study will lead to overestimation (underestimation) of the genotype frequency, which in forensic examination will lead to an unintended overestimation of the reliability level of the study.

**Table 3.**
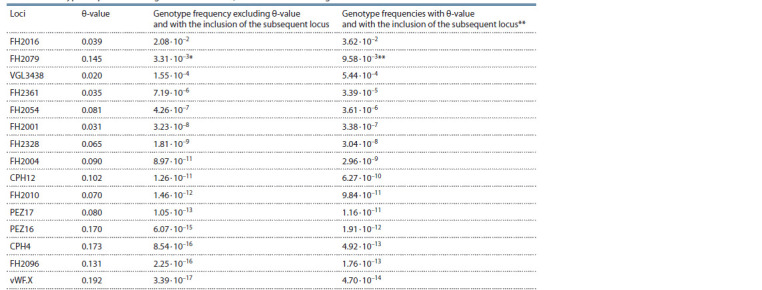
Genotype frequencies averaged over all animals, without and with using the θ-value The loci are listed in the order of increasing FST. * Here and below the product of the genotype frequency of the previous and current loci; ** here and below the product of the genotype frequency of the previous and current loci.

The analysis of PIC measures by Botstein et al. (1980) revealed that all selected loci in the total sample of dogs are highly informative for the study of the DNA of both the domestic dog and the Grey wolf (Table 4).

**Table 4.**
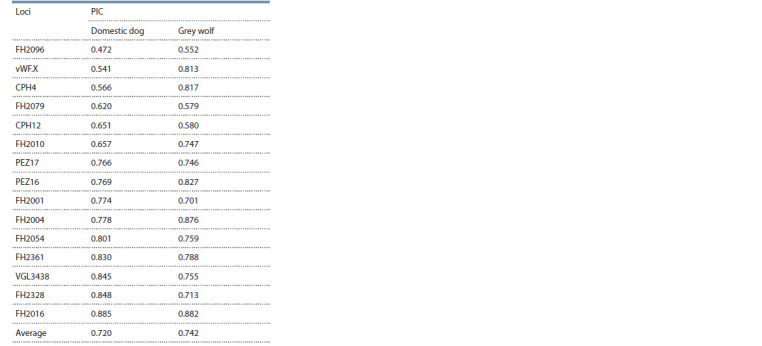
Values of the polymorphism information content of the selected loci for the samples of the Grey wolf and the domestic dog PIC – polymorphism information content of a locus.

In the combined sample of dogs, the minimum PIC value (0.472) was found at locus FH2096. The maximum PIC values were observed at FH2016 for the wolf (0.882) and at FH2016 for the dog (0.885). The average PIC values were 0.720 for the dog and 0.742 for the wolf, which can be considered significant for interpretation of results in a forensic genetic inves-tigation.

While the allele sequences of the wolf and dog were identical, allele sequencing revealed the presence of simple repeats. Incomplete tandem repeats were identified in the alleles of the FH2016, FH2361, and FH2328 loci. Specifically, for the FH2016 and FH2328 loci, incomplete tandems were detected both in the sample of wolves and in the sample of dogs. For the FH2361 locus, microvariants were identified only in the sample of dogs.

**Fig. 4.**
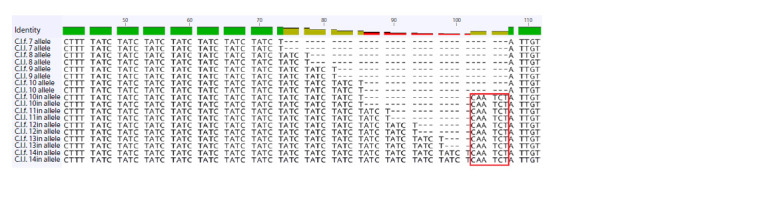
Part of the sequence of the FH2001 locus in DNA of the dog and the wolf. C.l.f. – domestic dog, C.l.l. – grey wolf. Red rectangle – 6 bp insertion in the non-tandem region of the locus.

Sequencing of the alleles of the FH2001 locus produced an unexpected result (Fig. 4). A 6 bp insertion located in the non-tandem region of the locus was found in the combined sample of wolves and dogs. This insertion was observed only in long alleles (with 10 or more tandem repeats), and there were no alleles with 11 or more repeats that did not contain insertions. The FH2001 locus was not excluded from the final forensic panel, and special names were assigned to alleles with insertion (“10in”–“14in” alleles).

Based on the results, we created the CPlex test system, which contains 15 STR loci and two sex loci. We obtained the nucleotide sequences of all the identified alleles; these alleles were identified in the tandem format for compatibility of the panel with the instrumentation. The sequences were deposited in the GenBank database (Table 5).

**Table 5.**
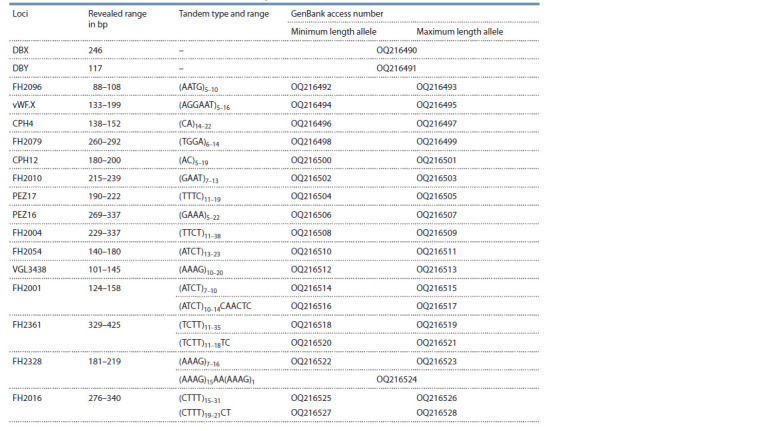
Characterization of microsatellite loci of the CPlex test system

The developed test system was validated in accordance with the protocol of the Scientific Working Group on DNA Analysis Methods (R.V. Guideline, 2004), and it was tested on benchmark samples and on real forensic objects. These methodological developments are being successfully used in expert practice when investigating the facts of illegal hunting, cruelty to animals and other offenses in the Republic of Belarus

## Conclusion

In this study, we selected 15 microsatellite loci and two sex loci that are suitable for forensic DNA examination of both the Grey wolf and the domestic dog. Furthermore, we designed a universal CPlex test system for the identification of individuals of the C. lupus species. Finally, we carried out an analysis of polymorphism and forensic parameters of loci and studied the genetic structure of the Belarusian populations of the C. lupus species. According to the results of statistical analysis of the dog and wolf genotype pools, the selected loci conform to the Hardy–Weinberg equilibrium. The coefficients of subdivision of the population for each STR locus of the test system were calculated, and the effectiveness of their use was proven. The developed CPlex test system was validated in accordance with the international standard and used in forensic research of cases of illegal hunting, animal attacks of people and livestock, as well as cases of cruelty to animals.

Based on the developed test system, we created two guides for practicing forensic experts, “The method of DNA-based identification of biological samples of the Grey wolf (Canis lupus lupus) and domestic dog (Canis lupus familiaris)” and “The method of using the computational tool for the analysis of genetic data of animals of the biological species Canis lupus – the Grey wolf (Canis lupus lupus) and the domestic dog (Canis lupus familiaris)”. The computational tool contains arrays of genotypes and a mathematical apparatus that allows one to automate the analysis of data when identifying species of the Grey wolf or the domestic dog, calculating the reliability of an expert conclusion in an identification study or establishing biological relationship; it also allows conducting DNA fingerprinting registration of biological samples of animals of C. lupus species in a forensic examination. The developed methods are included in the Register of forensic methods and other methodological materials of the State Forensic Examination Committee of the Republic of Belarus, which constitutes the implementation of the development in the national legal system.

## Conflict of interest

The authors declare no conflict of interest.
